# Nicorandil in preventing contrast-induced nephropathy in patients undergoing cardiac catheterization procedures: a systematic review and meta-analysis

**DOI:** 10.1097/MS9.0000000000003487

**Published:** 2025-07-02

**Authors:** Ramsha Waseem, Ajay Kumar, Simran Kumari, Sagar Kumar, Deepa Bai, Ganesh Kumar, Maheen Jabbar, Taimoor Ashraf, FNU Nancy, Bibi Mariam, Shah Dev, Muskan Turesh, Muhammad Hamza Yousuf, Umer Ejaz, Sayed Jawad

**Affiliations:** a Department of Medicine, Shaheed Mohtarma Benazir Bhutto Medical College Lyari, Karachi, Pakistan; b Department of Medicine, Ghulam Muhammad Mahar Medical College, Sukkur, Pakistan; c Department of Medicine, Bahria University Medical and Dental Collage, Karachi, Pakistan; d Department of Medicine, People’s University of Medical & Health Sciences for Women, Pakistan; e Department of Medicine, Nishtar Medical University Multan, Pakistan; f Department of Medicine, Chandka Medical College, Larkana, Pakistan; g Department of Medicine, Dow University of Health Sciences, Karachi, Pakistan; h Department of Medicine, Rawalpindi Medical University, Rawalpindi, Pakistan; i Department of Medicine, Kabul University of Medical Sciences, Kabul, Afghanistan

**Keywords:** coronary angiography, creatinine, cystatin C, humans, nicorandil, percutaneous coronary intervention

## Abstract

**Background:**

Contrast-induced nephropathy (CIN) remains a significant complication in patients undergoing coronary angiography (CAG) and percutaneous coronary intervention (PCI).

**Methods:**

A comprehensive literature search was conducted across PubMed, MEDLINE, Embase, Google Scholar, and Web of Science up to May 2024 to identify randomized controlled trials (RCTs) evaluating the efficacy and safety of nicorandil in patients undergoing CAG or PCI. The primary outcome was CIN incidence, while secondary outcomes included, changes in serum creatinine, serum cystatin C, blood urea nitrogen (BUN), and estimated glomerular filtration rate (eGFR). Risk ratios (RRs) and standardized mean differences (SMDs) with corresponding 95% confidence intervals (CIs) were pooled using a random-effects model. Heterogeneity was assessed using the I^2^ statistic.

**Results:**

Eleven RCTs and one prospective cohort study involving 2910 patients were included. Nicorandil administration was associated with a significant reduction in CIN incidence (RR: 0.40 [0.31–0.52], *P* < 0.00001), with both oral (RR: 0.35 [0.25–0.48], *P* < 0.00001) and intravenous formulations (RR: 0.52 [0.30–0.92], *P* = 0.02) demonstrating efficacy (p-interaction = 0.22). Patients receiving nicorandil exhibited significantly lower serum creatinine levels at 48 hours (SMD: −0.34 [–0.52, −0.16], *P* = 0.0002) and 72 hours (SMD: −0.24 [–0.40, −0.08], *P* = 0.003) post-procedure. Serum cystatin C was also significantly reduced at 48 hours (SMD: −0.48 [–0.81, −0.15], *P* = 0.004). However, nicorandil did not produce a significant change in eGFR at 24 hours (SMD: 0.17 [–0.07, 0.41], *P* = 0.17), 48 hours (SMD: 0.13 [–0.10, 0.37], *P* = 0.26), or 72 hours (SMD: 0.19 [–0.07, 0.45], *P* = 0.36).

**Conclusion:**

Nicorandil administration reduces CIN incidence and improves renal biomarker profiles in patients undergoing CAG and PCI. Further large-scale trials are necessary to validate its renoprotective properties.

## Introduction

Over a million cardiac catheterization procedures are performed in the United States (US) every year, primarily for diagnosing and treating coronary heart disease (CHD)^[1]^. Cardiac catheterization can be diagnostic or therapeutic, involving both right and left heart procedures^[2]^. While major complications are rare, acute kidney injury (AKI) due to contrast-induced nephropathy (CIN) remains a significant concern, especially in high-risk groups, where its incidence can reach as high as 20% to 30%^[3,4]^. Recent studies have shown that CIN is associated with long-term adverse events and mortality^[5,6]^. The 2011 European Society of Urogenital Radiology (ESUR) guidelines define CIN as a 0.5 mg/dL (44.2 µmol/L) increase in serum creatinine or more than 25% within 72 hours post-intravascular iodine contrast injection, excluding other influencing factors like surgery or nephrotoxic drugs^[7]^.

Although several treatment approaches have been studied to prevent CIN^[8]^, including hydration^[9]^, N-acetylcysteine (NAC)^[10]^, high-dose statin therapy^[11]^, sodium glucose co-transport 2 inhibitors (SGLT2) inhibitors^[12]^, Vitamin C, and theophylline^[13,14]^ which target pro-inflammatory cascades and relieve oxidative stress^[15,16]^, no definitive conclusions have been made regarding their efficacy and safety^[17–20]^. Nicorandil, an ATP-sensitive potassium channel activator and nitric oxide donor, exhibits vasodilatory effects and has been widely used as an anti-anginal agent^[21,22]^. Nicorandil is also known to suppress the TLR4/MAPK and P38/NF-κb/TNF-α inflammatory signaling cascade, potentially offering renoprotection against CIN^[23]^. Due to its vasodilatory effects and antioxidant properties, nicorandil can potentially help mitigate ischemia-reperfusion injury and CIN.

The efficacy and safety of oral and intravenous nicorandil in preventing CIN in patients undergoing cardiac catheterization procedures have remained unclear due to the lack of reliable evidence and contrasting findings in prior clinical trials and meta-analyses due to the lack of statistical power. In light of these inconsistent results and with the publication of recent randomized controlled trials, we conducted an updated meta-analysis to assess the efficacy and safety of nicorandil in reducing the risk of CIN and its renoprotective effects in terms of its impact on post-intervention serum creatinine, cystatin levels, blood urea nitrogen (BUN), and the estimated glomerular filtration rate (eGFR).

## Methods

This systematic review and meta-analysis was conducted in accordance with Preferred Reporting Items for Systematic Review and Meta-analysis (PRISMA) guidelines^[24]^. A thorough electronic search, without language limitations, was conducted on PubMed, Embase, and the Cochrane Central Register of Controlled Trials from inception through May 2024 using a predefined search strategy (Supplemental Digital Content Table S1, available at: http://links.lww.com/MS9/A858). Reference lists of retrieved trials, previous meta-analyses, and review articles were manually screened to identify relevant studies. We used a comprehensive search strategy including terms such as “nicorandil,” “contrast-induced nephropathy,” “contrast media,” and “cardiac catheterization,” using Boolean operators (AND, OR) and appropriate filters for study type. Gray literature was searched through ClinicalTrials.gov and WHO ICTRP. The eligibility criteria included (1) published randomized controlled trials or prospective cohort studies comparing outcomes in patients receiving nicorandil with those receiving control or placebo, (2) involving adult males or females (>18 years) undergoing PCI or CAG, and (3) with outcomes reported as contrast-induced nephropathy or serum creatinine levels. Case reports, review articles, gray literature, and non-English language articles were excluded.

Results were imported into EndNote for bibliographic management^[25]^, and all duplicate citations were eliminated. Two independent reviewers (MZT and H) meticulously evaluated titles, abstracts, and full-text articles of the remaining articles and shortlisted those that met the predefined eligibility criteria. Any discrepancies were resolved through consultation with a third investigator (MSA). Outcomes of interest included the incidence of CIN, post-intervention serum creatinine levels, cystatin C, blood urea nitrogen (BUN), estimated glomerular filtration rate (eGFR) levels at 24 hr, 48 hr, and 72 hr intervals. The Cochrane Risk of Bias tool^[26]^ was used for quality assessment of the RCTs while the Newcastle-Ottawa Scale (NOS)^[27]^ was used for the prospective cohort.

### Statistical analysis

Statistical analyses were conducted using Review Manager version 5.3. Results were presented as risk ratios (RRs) with 95% confidence intervals (CIs) and pooled using a random effects model. For continuous outcomes (e.g., serum creatinine, BUN, cystatin C), we used standardized mean difference (SMD) with 95% confidence intervals. Forest plots were used for visual representation of the pooled results, with subgroup differences assessed using the Chi-square test. Higgins I^2^ was used to evaluate heterogeneity, which was deemed acceptable if it was less than 50%^[28]^. Publication bias was assessed using funnel plot inspection, with further statistical analysis using Begg’s and Egger’s tests^[29]^. A *P* value less than 0.05 was considered significant in all cases. A sensitivity analysis was performed to assess the impact of excluding studies at high risk of bias.

**HIGHLIGHTS**

- Nicorandil significantly reduces the incidence of contrast-induced nephropathy (CIN) in patients undergoing coronary angiography (CAG) and percutaneous coronary intervention (PCI).
- Nicorandil lowers serum creatinine and serum cystatin C levels, indicating improved renal function post-procedure.
- Large-scale randomized controlled trials are needed to confirm its long-term renoprotective effects and optimal administration strategy.

## Results

### Literature search

**Figure 1.**
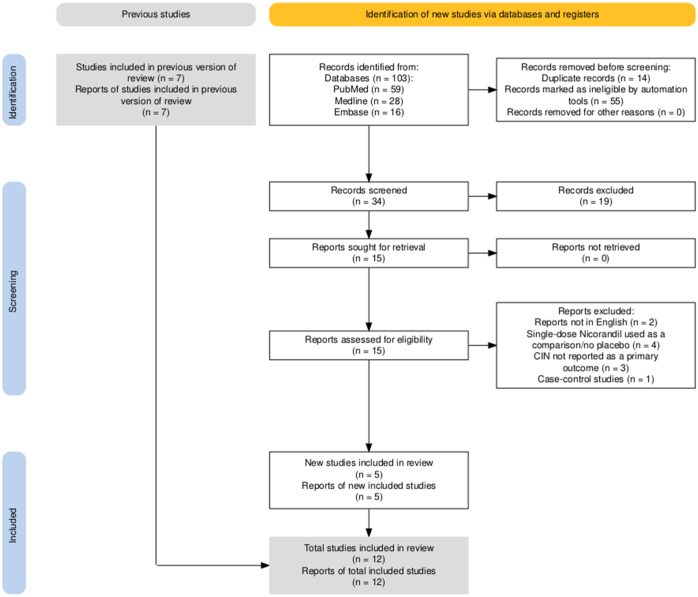
PRISMA flow diagram.

Of the 103 articles retrieved from the initial search, 12 studies (n = 2910 patients) were selected for inclusion in the final analysis. The PRISMA flow chart summarizes the detailed search and study selection process (Fig. 1).

### Characteristics of included studies

Of the 12 included studies, 11 were RCTs^[30–40]^, and one was a prospective cohort study^[41]^. The studies included a total of 2910 patients who were undergoing cardiac catheterization, with 1454 patients in the nicorandil group and 1456 in the placebo group assigned randomly. In eight studies, nicorandil was given orally^[30,32,33,35–37,39,40]^, while in the other four studies, the intravenous formulation of nicorandil was injected^[31,34,38,41]^. The included studies enrolled a total of 2910 patients, comprising 1906 male and 1025 female participants. The included studies enrolled adult patients undergoing CAG or PCI, with varying degrees of renal function. Some studies included patients with mild to moderate chronic kidney disease or those at high risk of CIN, while others excluded patients with severe renal impairment or end-stage renal disease. Nicorandil administration protocols varied across studies, with doses ranging from 5 mg to 20 mg and routes including both intravenous and oral formulations. Baseline characteristics of the included studies are outlined in Table 1 in detail.

**Table 1:** Baseline characteristics

	Ko *et al*^[31]^	Nawa *et al*^[38]^	Fan *et al*^[36]^	Iranirad *et al*^[37]^	Fan *et al*^[30]^	Abd Elrahman *et al*^[32]^	Moghaddam *et al*^[33]^	Zhang *et al*^[42]^	Zhang X *et al*^[39]^	Geng *et al*^[43]^	Zeng *et al*^[41]^	Yusuf *et al*^[35]^
Year	2013	2015	2016	2017	2019	2023	2023	2019	2019	2023	2019	2024
Region	Seoul, Korea	Gifu, Japan	Xingtai, China	Qom, Iran	Beijing, China	Cairo, Egypt	Mashhad, Iran	Tianjin, China	Tianjin, China	Beijing, China	Tianjin, China	New Delhi, India
Study Population												
*Control*	76	107	120	64	125	200	172	125	150	113	112	105
Nicorandil	73	106	120	64	127	200	172	125	150	113	107	105
Age (years)												
*Control*	69.1 ± 10.3	70.1 ± 8.1	67.37 ± 6.33	57.64 ± 12.42	65.87 ± 17.62	59.7 ± 5.8	60.61 ± 10.17	67.0 ± 7.2	67.11 ± 7.19	58 ± 10	66.69 ± 7.33	60.46 ± 8.175
Nicorandil	70.8 ± 9.6	70.4 ± 7.7	66.07 ± 6.37	61.35 ± 11.77	62.25 ± 16.63	60 ± 8.7	60.97 ± 9.86	67.4 ± 6.6	67.25 ± 6.42	60 ± 11	67.09 ± 6.85	60.33 ± 8.731
Male (%)												
*Control*	51(67.1)	74 (78.7)	95 (79.17)	40 (62.5)	67 (53.60)	80 (40)	NR	89(71.2)	114 (76.0)	98 (86.7)	67 (39.8)	87(82.9)
Nicorandil	53(72.6)	80 (81.6)	88 (73.33)	39 (60.9)	76 (59.84)	87 (43.5)	NR	93 (74.4)	118 (78.7)	95 (84.1)	73 (68.2)	92(87.6)
BMI												
*Control*	24.8 ± 3.7	23.5 ± 2.9	22.28 ± 2.98	27.78 ± 4.8	23.78 ± 5.98	NR	27.31 ± 4.33	25.1 ± 2.0	25.10 ± 2.02	NR	24.60 ± 3.34	NR
Nicorandil	24.1 ± 3.2	23.4 ± 3.4	22.36 ± 2.19	28.43 ± 5.6	24.35 ± 5.87	NR	27.19 ± 4.25	24.9 ± 2.2	24.80 ± 2.17	NR	24.85 ± 2.63	NR
HTN (%)												
*Control*	61(80.3)	68 (72.3)	74 (61.67)	41 (64.1)	62 (49.6)	84 (42.0)	111 (64.53)	NR	71 (47.3)	64 (56.6)	59 (52.7)	44 (41.9)
Nicorandil	57(78.1)	69 (70.4)	69 (57.50)	35 (54.7)	68 (53.54)	103 (51.5)	115(66.86)	NR	69 (46.0)	57 (50.4)	69 (64.5)	30(28.6)
DM (%)												
*Control*	42(55.3)	47 (50.0)	62 (51.67)	26 (40.6)	75 (60)	73 (36.5)	107 (62.21)	29 (23.2)	35 (23.3)	21 (18.6)	18 (16.1)	41 (39.0)
Nicorandil	30(41.1)	57 (58.2)	66 (55.00)	27 (42.2)	81 (63.78)	83 (41.5)	99 (57.55)	24 (19.2)	34 (22.7)	29 (25.7)	21 (19.6)	37 (35.2)
LVEF (%)												
*Control*	(≤45%) 5 (6.6)	NR	51.15 ± 6.36	49.14 ± 5.8	53.58 ± 12.77	55.8 ± 7.5	50.78 ± 8.96	NR	60.10 ± 6.88	NR	NR	46.57 ± 11.547
Nicorandil	(≤45%)11 (15.1)	NR	50.36 ± 5.29	48.87 ± 6.8	51.39 ± 10.35	55.5 ± 6.1	50.74 ± 9.22	NR	60.11 ± 7.77	NR	NR	45.48 ± 11.509
eGFR mL/min/1.73 m^2												
*Control*	40.1 ± 13.9	58.1 ± 16.4	50.38 ± 5.74	83 ± 28.1	61.75 ± 22.56	NR	83.54 ± 28.81	51.0 ± 3.8	NR	NR	81.42 ± 26.84	60.26 ± 10.781
Nicorandil	37.5 ± 13.4	59.6 ± 16.5	49.62 ± 5.38	76.39 ± 24.6	59.32 ± 19.31	NR	78.91 ± 28.65	51.2 ± 4.1	NR	NR	77.41 ± 16.82	63.64 ± 9.088
Baseline SCr (mg/dL) OR mol/L												
*Control*	1.61 ± 0.44 mg/dL	1.02 ± 0.35 mg/dL	122.99 ± 10.39 µmol/L	1.0359 ± 0.15 mg/dl	118.75 ± 26.18 µmol/L	1.111 ± 0.31 mg/dL	80.44 ± 22.10	121.6 ± 15.1 µmol/L	123.54 ± 14.37(μmol/L)	82 ± 23(μmol/L)	76.62 ± 14.46(μmol/L)	1.323 ± 0.1815 mg/dL
Nicorandil	1.73 ± 0.60 mg/dL	0.99 ± 0.29 mg/dL	123.55 ± 10.77 µmol/L	1.0859 ± 0.22 mg/dl	121.22 ± 22.35 µmol/L	1.064 ± 0.34 mg/dL	83.98 ± 21.22	122.0 ± 16.0 µmol/L	124.00 ± 15.16(μmol/L)	80 ± 22(μmol/L)	78.46 ± 9.95(μmol/L)	1.268 ± 0.1605 mg/dL
Hydration (ml)												
*Control*	1.1 mL/kg/h	NR	849 ± 158	NR	1 mL/kg/h	NR	1.0 mL/kg/hr	1.0 mL/kg/hr	1.0 mL/kg/hr	NR	1.0 mL/kg/h,	1.0 mL/kg/h
*Nicorandil*	1.0 mL/kg/h	NR	837 ± 162	NR	1 mL/kg/h	NR	1.0 mL/kg/hr	1.0 mL/kg/hr	1.0 mL/kg/hr	NR	1.0 mL/kg/h,	1.0 mL/kg/h
Rate of contrast media infusion (mL/min)												
*Control*	126.9 ± 74.6	146.3 ± 63.6	149.2 ± 57.0	202.26 ± 44.4	122.81 ± 35.92	154.1 ± 54.1	NR	167.0 ± 46.7	144.50 ± 10.56	161 ± 20	184.79 ± 53.05	128 ± 55.598
Nicorandil	125.6 ± 69.1	135.2 ± 57.0	145.3 ± 51.6	213.98 ± 44.6	128.39 ± 37.25	162.6 ± 59.6	NR	166.4 ± 49.6	145.45 ± 10.68	159 ± 19	172.83 ± 49.66	118.57 ± 49.738
Hydration (ml)												
*Control*	1.1 mL/kg/h	NR	849 ± 158	NR	1 mL/kg/h	NR	1.0 mL/kg/hr	1.0 mL/kg/hr	1.0 mL/kg/hr	NR	1.0 mL/kg/h,	1.0 mL/kg/h
*Nicorandil*	1.0 mL/kg/h	NR	837 ± 162	NR	1 mL/kg/h	NR	1.0 mL/kg/hr	1.0 mL/kg/hr	1.0 mL/kg/hr	NR	1.0 mL/kg/h,	1.0 mL/kg/h
Rate of contrast media infusion (mL/min)												
*Control*	126.9 ± 74.6	146.3 ± 63.6	149.2 ± 57.0	202.26 ± 44.4	122.81 ± 35.92	154.1 ± 54.1	NR	167.0 ± 46.7	144.50 ± 10.56	161 ± 20	184.79 ± 53.05	128 ± 55.598
Nicorandil	125.6 ± 69.1	135.2 ± 57.0	145.3 ± 51.6	213.98 ± 44.6	128.39 ± 37.25	162.6 ± 59.6	NR	166.4 ± 49.6	145.45 ± 10.68	159 ± 19	172.83 ± 49.66	118.57 ± 49.738

### Effect of nicorandil on the incidence of contrast-induced nephropathy

**Figure 2.**
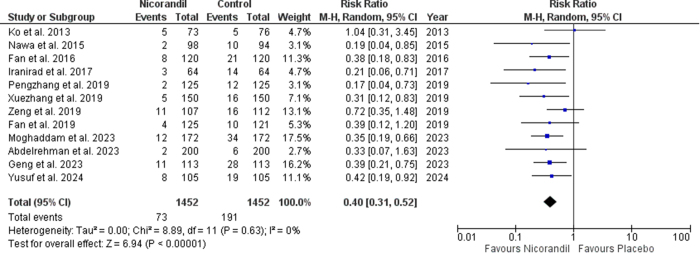
Incidence of CIN.

**Figure 3.**
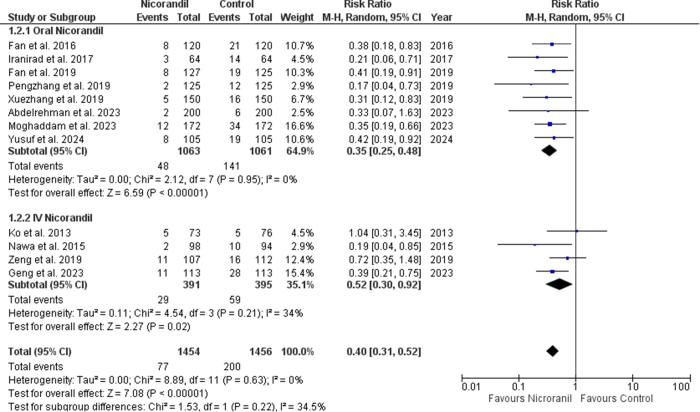
CIN by subgroups of IV and oral route.

Our results showed that Nicorandil reduced the incidence of CIN in patients undergoing cardiac catheterization significantly (RR 0.40 [0.31, 0.52]; *P* < 0.00001; I^2^ = 0%) (Fig. 2). The funnel plot was asymmetrical, indicating a potential risk of publication bias, which was ruled out through Begg’s and Egger’s tests (Supplemental Digital Content Figure S1, available at: http://links.lww.com/MS9/A858). Subgroup analysis based on the route of administration showed trends toward better efficacy upon intravenous administration of nicorandil (RR 0.52 [0.30, 0.92]; *P* = 0.02; I^2^ = 34%) as compared to oral nicorandil (RR 0.35 [0.25, 0.48]; *P* < 0.00001; I^2^ = 0%), however this subgroup difference was non-significant (p-interaction = 0.22) in preventing CIN in patients undergoing cardiac catheterization (Fig. 3).

### Effect of nicorandil on serum creatinine levels

**Figure 4.**
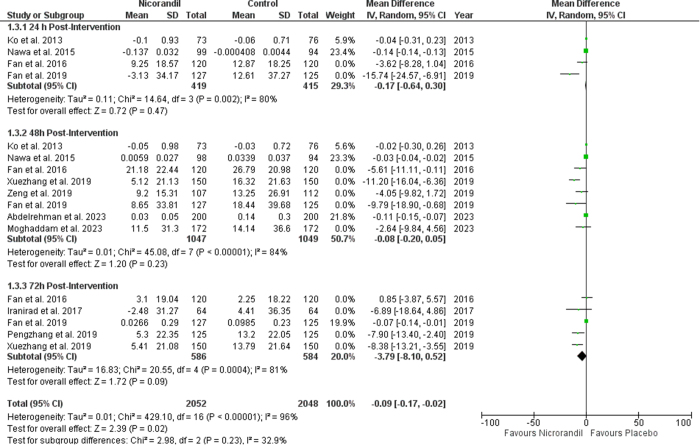
Change in serum creatinine.

Serum creatinine levels were significantly lower at 48 hours post-intervention in the nicorandil group compared to the control group (SMD −0.34 [−0.52, − 0.16], *P* = 0.0002; I^2^ = 76%). The levels were still lower 72 hours post-intervention (SMD −0.24 [−0.40, −0.08], *P* = 0.003; I^2^ = 47%). Serum creatinine levels 24 h hour post intervention did not change significantly in patients receiving nicorandil as compared to those receiving a placebo drug (SMD −1.04 [−2.23, + 0.14], *P* = 0.08; I^2^ = 98%). Sub-group analysis based on post-intervention follow-up times showed no significant difference between subgroups (*P* value for subgroup difference = 0.33) (Fig. 4).

### Effect of nicorandil on serum cystatin levels

**Figure 5.**
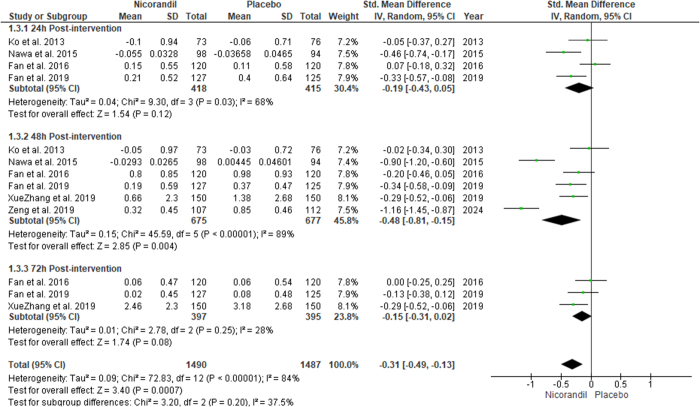
Change in serum cystatin C levels.

Our results showed that serum cystatin levels were significantly lower 48 hours post-intervention in the Nicorandil group compared to the control group (SMD −0.48 [−0.81, −0.15], *P* = 0.004; I^2^ = 89%). However, the results were non-significant for serum cystatin levels at 24 h (SMD −0.19 [−0.43, 0.05], *P* = 0.12; I^2^: 68%) and 72 h (SMD −0.15 [−0.31, 0.02], *P* = 0.08; I^2^: 28%) post-intervention. Sub-group analysis based on post-intervention times showed no significant difference between subgroups (*P* value for subgroup difference = 0.20) (Fig. 5).

### Effect of nicorandil on BUN

**Figure 6.**
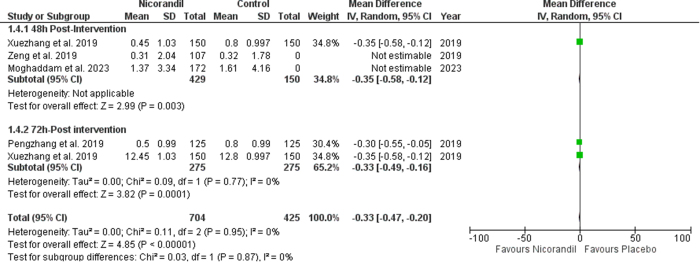
Change in BUN.

Compared to the control, nicorandil had no significant effect on post-intervention BUN levels (SMD −0.14 [−0.35, 0.06], *P* = 0.17; I^2^ = 56%) 48 hours post-intervention. However, BUN levels were significantly lower 72 hours post-intervention (SMD −0.33 [−0.49, 0.16], *P* = 0.0002; I^2^ = 0%) in patients receiving nicorandil as compared to controls (Fig. 6). Sub-group analysis based on post-intervention times showed no significant difference between subgroups (*P* value for subgroup difference = 0.17).

### Effect of nicorandil on eGFR

**Figure 7.**
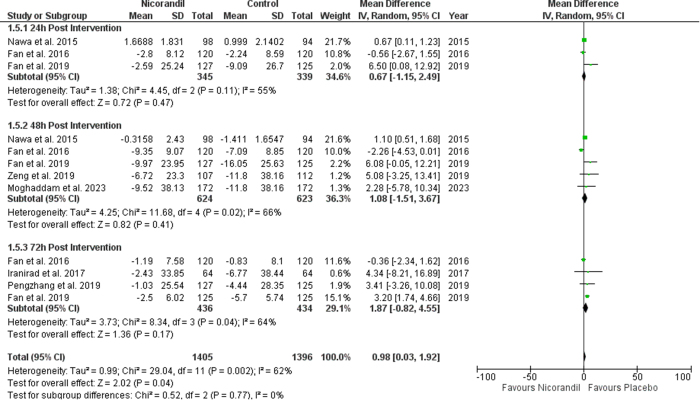
Change in eGFR.

The results showed a non-significant effect of nicorandil compared to placebo on eGFR values at 24 hours (SMD 0.17 [−0.07, 0.41], *P* = 0.17; I^2^ = 60%), 48 hours (SMD 0.13 [−0.10, 0.37], *P* = 0.26; I^2^ = 76%), and 72 hours (SMD 0.19 [−0.07, 0.45], *P* = 0.16; I^2^ = 73%). There was no significant difference between subgroups (*P* value for overall effect = 0.95) (Fig. 7).

### GRADE qualification

The Grading of Recommendations, Assessment, Development, and Evaluations (GRADE) qualification showed a high level of certainty for the reduction of using nicorandil. According to the Grading of Recommendations, Assessment, Development, and Evaluations (GRADE)^[44]^ assessment, the certainty of evidence supporting the reduction of contrast-induced nephropathy with nicorandil was rated as high. Assessment of publication bias using both Begg’s test (*P* = 0.1702), and Egger’s regression (*P* = 0.2709) showed statistically non-significant results (Supplemental Digital Content Figure S1, available at: http://links.lww.com/MS9/A858).

### Risk of bias assessment

Upon risk of bias assessment, studies were deemed to have a moderate risk of bias due to the high risk of blinding of participants and personnel (performance bias) due to the open-label nature of the studies. The risk of bias assessment for the studies is presented in Supplemental Digital Content, Figure S2 (available at: http://links.lww.com/MS9/A858). Risk of bias assessment for the observational study included is presented in Supplemental Digital Content, Table S2 (available at: http://links.lww.com/MS9/A858). Sensitivity analysis excluding high-risk studies did not significantly alter the pooled effect sizes, supporting the robustness of the findings.

## Discussion

In this meta-analysis assessing the efficacy and safety of nicorandil among patients following CAG and PCI procedures, we report several key findings. First, treatment with nicorandil reduced the incidence of CIN, with oral administration showing significantly better efficacy compared to intravenous administration in reduction of CIN (4.5% vs. 7.4%, *P* = 0.04). Second, nicorandil consistently reduced post-intervention serum creatinine and serum cystatin levels without significantly affecting eGFR. These findings are essential because CIN is a significant complication of cardiac catheterization procedures, potentially leading to permanent kidney damage, increased mortality, and a substantial burden on the healthcare system^[45]^. Nicorandil’s demonstrated efficacy in reducing the risk of CIN suggests its potential as a proactive renal protection strategy in high-risk patient populations. This improves patient safety and reduces the burden of renal complications. Moreover, by optimizing treatment protocols to include nicorandil alongside hydration and other renal protective measures, clinicians may enhance patient outcomes while potentially mitigating healthcare costs associated with managing CIN-related complications.

Our study offers several contributions to the literature. It includes the largest number of trials (n = 12) to date, expanding the evidence base to 2910 patients – the highest of any prior meta-analysis on this topic. This not only increases the statistical power of our findings but also strengthens their generalizability. Moreover, we incorporated recently published trials (2023–2024) and analyzed outcomes not previously synthesized in this context, including serum cystatin C, blood urea nitrogen (BUN), and eGFR. Additionally, our subgroup analysis based on nicorandil administration route (oral vs. intravenous) provides novel insights into route-specific effects. These aspects distinguish our study and provide incremental value for clinicians seeking to understand the full scope of nicorandil’s renoprotective effects in cardiac catheterization procedures. While some studies lacked blinding, the influence of performance bias is likely limited due to the objective nature of the primary outcomes. Furthermore, sensitivity analysis excluding high-risk studies did not materially affect results. The heterogeneity observed in some outcomes, particularly serum creatinine and cystatin C, may stem from methodological differences among studies. These include variations in nicorandil dosage (ranging from 5 mg to 20 mg), differences in administration route (oral vs. intravenous), and diverse baseline renal function among study populations. Additionally, inconsistencies in the timing of outcome measurement (e.g., 24, 48, or 72 hours) and follow-up duration may contribute to the between-study variability. These differences highlight the clinical and methodological diversity that underpins the moderate to high statistical heterogeneity seen in some outcomes. Although subgroup analyses were conducted, heterogeneity persisted due to methodological differences across trials. This variability limits the certainty of pooled estimates and should be considered when interpreting the findings.

Our pooled analysis revealed a significant protective effect of nicorandil against CIN in patients undergoing cardiac catheterization procedures. These findings align with those of Pranata *et al*^[42]^. However, it is important to note that these findings are contradictory to the results of individual studies by Ko *et al*^[31]^, Zeng *et al*^[41]^, and Abd Elrahman *et al*^[32]^. The discrepancy between our findings and those of Ko *et al*^[31]^ may be attributed to the fact that Ko *et al* administered nicorandil before performing the procedure that could have led to its rapid washout and potentially limited its protective effects against CIN. The shorter half-life of nicorandil (approximately 1 hour) compared to contrast agents (up to 2 hours and up to 4 hours in individuals with impaired renal function) suggests that prolonged use of nicorandil after PCI may be more effective in reducing the risk of kidney injury^[32]^.

The subgroup analysis confirmed better efficacy of oral nicorandil in reducing CIN incidence compared to intravenous use (*P* = 0.04), although further head-to-head trials are needed to validate these findings. The findings of Geng 2023^[34]^ and Zeng 2024^[41]^, suggest that the IV route is superior to the oral route for nicorandil administration in patients. IV nicorandil provides better renal protection by ensuring higher and more consistent plasma levels of the drug, leading to improved renal perfusion and reduced oxidative stress^[43]^. This is particularly important for patients with pre-existing kidney conditions or other risk factors for CIN. Since the incidence of CIN was consistent across most of the studies included in our meta-analysis, the results of this meta-analysis are more generalizable to different patient populations and clinical settings. This increases the applicability of the findings and informs clinical practice. The apparent superior efficacy of oral nicorandil over intravenous formulations may be partly explained by pharmacokinetic differences, where oral administration achieves more prolonged therapeutic levels. Alternatively, selection bias could have influenced this finding, as trials using intravenous nicorandil often enrolled higher-risk patients, potentially attenuating observable benefits. These interpretations should be considered exploratory and warrant further investigation in head-to-head comparisons.

One postulated mechanism by which nicorandil prevents kidney damage is by suppressing inflammatory pathways and increasing renal blood flow^[23]^. Our study found that nicorandil significantly lowered the plasma creatinine and cystatin levels without altering the eGFR. This indicates that nicorandil can potentially improve the clearance of toxic metabolites without compromising the integrity of the filtration barrier. Additionally, nicorandil was also found to decrease the risk of major adverse effects (MAEs), including cardiovascular (cardiac arrest, MI, worsening of heart failure/acute HF, ventricular fibrillation, malignant arrhythmias, emergency coronary bypass graft, emergency PCI for acute thrombosis, stroke and minor bleeding), renal (end-stage kidney disease, patients having to undergo dialysis), gastrointestinal (upper GI bleeding) and headache. This attenuated risk may positively impact the patient’s quality of life and treatment adherence. Previous meta-analysis^[42]^ had suggested a decrease in the incidence of CIN. However, our study augmented the validity of the evidence by adding about five more studies and using markers like serum creatinine and cystatin for a precise estimation of the extent of kidney functionality.

Compared to other pharmacological strategies such as statins, sodium bicarbonate, fenoldopam, natriuretic peptides, N-acetylcysteine, vitamins, theophylline, and prostaglandins, nicorandil has demonstrated favorable safety and efficacy in preventing CIN. A daily dose of 30 mg nicorandil is more effective than BNP (1.5 ug/kg) administered over 24 hours in reducing serum creatinine levels and CIN incidence^[46]^. For CIN prophylaxis, the effects of oral N-acetylcysteine have mainly been studied; its efficacy in emergency use is questionable. On the other hand, theophylline has primarily been used only as an adjunct medication along with IV hydration and other pharmacological interventions, i.e. sodium bicarbonate NAC. Different forms of prophylactic therapy require further studies to determine their employment in clinical practice and the standardization of their doses, as the existing literature yields inconsistent results across studies^[17–20]^.

### Limitations

It is essential to consider certain limitations while interpreting meta-analysis results. First, our meta-analysis included an observational study, which may have induced the risk of selection bias owing to the study’s design. Second, it is also important to acknowledge that most of our included studies were single-centered and lacked double-blinding, which may have led to an increased risk of observer bias in these studies. Third, most of these clinical trials were conducted in Asian countries (China, Iran, Korea, Japan, Egypt and India), which may limit the generalizability of their meta-analysis to the global population. Fourth, most of the included studies selected patients with mild to moderate renal dysfunction and excluded the patients with end-stage renal disease, which limits the extrapolation of their results to patients with a broader spectrum of the disease severity. Fifth, the heterogeneity in baseline renal function and nicorandil dosing strategies across included studies may contribute to between-study variability and should be considered when interpreting the pooled results. In cases of moderate to high statistical heterogeneity, interpretation should be guided by clinical variability among study populations, renal function baseline, and differences in nicorandil dosage and route. Although no a priori power analysis was performed given the meta-analytic design, the inclusion of 2910 participants across 12 studies provided sufficient statistical power for detecting clinically relevant effects. Lastly, since most of our included studies reported the effects of the intervention after short follow-up periods, the estimation of long-term treatment effects may be compromised.

## Conclusion

Nicorandil administration has the potential to reduce the risk of contrast-induced nephropathy (CIN) in CAG/PCI patients. It lowers plasma creatinine and Cystatin C levels by enhancing renal clearance without significantly affecting eGFR. Further large-scale trials with more extended follow-up periods are warranted to ascertain the renoprotective effects of nicorandil and its potential to mitigate the risks of major adverse events.

## Data Availability

All the data used in this study are publicly available in the trials, which are referenced in the bibliography.

## Research registration unique identifying number (UIN)

Name of the registry: National Institute for Health Research (NIHR) International prospective register of systematic reviews (PROSPERO), Unique Identifying number or registration ID: CRD2025653620, Hyperlink to your specific registration (must be publicly accessible and will be checked): ahttps://www.crd.york.ac.uk/prospero/display_record.php?ID=CRD42025653620.
